# Simultaneous Detection of Carbon Quantum Dots as Tracers for Interwell Connectivity Evaluation in a Pattern with Two Injection Wells

**DOI:** 10.3390/nano14090789

**Published:** 2024-05-01

**Authors:** Stephania Rosales, Karol Zapata, Farid B. Cortes, Benjamín Rojano, Carlos Diaz, Carlos Cortes, David Jaramillo, Adriana Vasquez, Diego Ramirez, Camilo A. Franco

**Affiliations:** 1 Grupo de Investigación en Fenómenos de Superficie–Michael Polanyi, Facultad de Minas, Universidad Nacional de Colombia, Sede-Medellín, Medellín 050034, Colombia; srosalesd@unal.edu.co (S.R.); kzapata@unal.edu.co (K.Z.); 2 Grupo de Investigación Química de los Productos Naturales y los Alimentos, Facultad de Ciencias, Universidad Nacional de Colombia, Sede-Medellín, Medellín 050035, Colombia; brojano@unal.edu.co; 3 GeoPark Colombia SAS, Bogotá 111211, Colombia; cdiaz@geo-park.com (C.D.); ccortes@geo-park.com (C.C.); 4 Verano Energy Limited Sucursal, Bogotá 110211, Colombiaadriana.vasquez@parexresources.com (A.V.);

**Keywords:** carbon quantum dots, fluorescence, interwell connectivity, simultaneous detection, tracers, waterflooding, nanotechnology

## Abstract

This study aimed to develop and implement a nanotechnology-based alternative to traditional tracers used in the oil and gas industry for assessing interwell connectivity. A simple and rapid hydrothermal protocol for synthesizing carbon quantum dots (CQDs) using agroindustry waste was implemented. Three commercial CQDs were employed (CQDblue, CQDgreen, and CQDred); the fourth was synthesized from orange peel (CQDop). The CQDs from waste and other commercials with spherical morphology, nanometric sizes less than 11 nm in diameter, and surface roughness less than 3.1 nm were used. These tracers demonstrated high colloidal stability with a negative zeta potential, containing carbonyl-type chemical groups and unsaturations in aromatic structures that influenced their optical behavior. All materials presented high colloidal stability with negative values of charge z potential between −17.8 and −49.1. Additionally, individual quantification of these tracers is feasible even in scenarios where multiple CQDs are present in the effluent with a maximum percentage of interference of 15.5% for CQDop in the presence of the other three nanotracers. The CQDs were injected into the field once the technology was insured under laboratory conditions. Monitoring the effluents allowed the determination of connectivity for five first-line producer wells. This study enables the application of CQDs in the industry, particularly in fields where the arrangement of injector and producer wells is intricate, requiring the use of multiple tracers for a comprehensive description of the system.

## 1. Introduction

For the hydrocarbon industry, it is essential to predict the flow of fluids through complex and deep geological formations during waterflooding and enhanced recovery processes. The ability to predict fluid behavior has significant implications for reservoir management and can help optimize production and recovery [1]. One strategy to accomplish this mission is to inject tagged fluids with discriminative tracers and monitor their appearance in producing wells. This methodology has demonstrated its efficacy in precisely delineating fluid displacement, heterogeneous reservoir structures, and impediments to fluid flow. Its application enables more intricate and accurate modeling of reservoir dynamics, thereby fostering the development of advanced strategies for field development [2]. A desirable behavior of the tracers is that they remain in the injected phase without interacting with the formation and the crude oil. Other tracers, the partitioned ones, can selectively display the signal just by the presence of oil [3]. They can indicate formation saturation and are particularly useful in cases where the injected fluid and crude oil are miscible or similar in composition. Combining these different types of tracers makes it possible to understand fluid behavior in the reservoir and optimize enhanced oil recovery strategies accordingly [4]. 

Tracers have been extensively employed in the oil and gas industry for 60 years. Their applications in water erosion processes, hydrogeological inquiries, geothermal investigations [5], and well connectivity determination [6] are among the most important and widespread tracer applications. Two predominant categories of tracers have been employed for these diverse applications: radioactive and chemical. Quantifying the application of these tracers in the oil and gas industry has been extensive due to their ability to comprehensively delineate subsurface and near-well properties effectively. Despite their prevalent usage, both exhibit limitations linked to their environmental footprint, the costs, and the technical intricacies involved in their precise quantification [7]. Tritiated water is a common radioactive tracer due to its similarity to normal water, making it useful for measuring fluid flow without disturbing the chemical equilibrium in most reservoirs [8]. Other tracers are based on radioactive isotopes of metal cations such as Co-57, Co-60, Cs-134, and Eu-154 [9]. Despite their utility, the radioactive nature of these compounds presents several inherent problems, including biotic toxicity and difficulties with transport, analysis instrumentation, and costs. However, they face significant environmental criticism. Additionally, their quantification requires complex analysis methods such as gas chromatography, high-performance liquid chromatography (HPLC), and liquid scintillation counting, making the process neither simple nor swift.

These factors have led to decreased interest in its use over time. As a result of this need, technological advances and the availability of alternative tracer options have helped to mitigate some of these problems [5]. Chemical compounds have replaced radioactive tracers due to their variety, lower toxicity, and ease of production [6]. Benzoic acid derivatives are commonly used to study interconnected wells. However, they have limitations related to high concentrations for accurate detection and their potential interaction with formation or oil [7]. Also, they are classified as recalcitrant and toxic to humans and animals [10]. As a result, complex and safety-intensive instrumental methods have been developed for their quantification, leading to higher operating costs and delays in obtaining results. These challenges can result in inappropriate decision-making in the field [11]. 

Nanotechnology-based tracers have been proposed for use in the oil and gas industry to overcome the difficulties related to conventional tracers. Significant laboratory-scale investigations were conducted to assess the stability of carbon quantum dots (CQDs) when exposed to metallic ions, high salt concentrations, pH fluctuations [12], and their behavior within porous media under both static and dynamic conditions [13]. These studies encompassed analyses of CQD movement within packed columns, fluorescence intensity correlation with CQD concentration, and simulation of CQD transport within sandstone cores saturated with oil using a modified 1-D advection-dispersion-reaction (ADR) equation [14]. The results obtained from these experiments unveiled remarkable dispersion characteristics in aqueous environments, nanoscale dimensions, outstanding optical properties, resilience to adverse conditions of salinity and temperature, and an extended operational lifespan [15]. Notably, during the experiment, the recovery of CQDs after displacement through a low-permeability core approached 76% in a water solution with one wt% NaCl at 80 °C. These laboratory findings collectively demonstrate the favorable performance of CQDs and suggest the potential for their scalable utilization.

The application of CQDs as tracers has exhibited promising outcomes at the laboratory scale, yet field implementations have remained limited. Kanj et al. [16] evaluated specifically synthesized and functionalized CQDs called “A-Dots”. Examination of these tracers revealed detection thresholds within the parts per billion range. Deployment in an observation well, utilizing a “huff-and-puff” scheme, allowed for a two-day sampling period, resulting in an impressive recovery rate exceeding 86% of the CQDs. Alkasar et al. [17] employed A-Dots as tracers in a carbonate reservoir, where they were injected at an approximate 3000 mg/L concentration. These tracers were identified in producing wells for 60 days post-injection, indicating that the A-Dots exhibited an earlier appearance compared to conventional chemical tracers. In another study, Franco et al. [15] synthesized carbon quantum dots derived from Mortiño (*Vaccinium Meridionale Swartz*) extract and used them as tracers in a sandstone formation under realistic laboratory and field conditions. The authors injected the obtained nanomaterials at a concentration of 500,000 mg/L in Colombia. The evaluation of these tracers spanned ten producing wells, revealing diverse interconnections within the field. The study observed tracer bursts occurring in certain wells three days post-injection, persisting for an additional 15 days. The initial occurrence was noted in another well after three days, enduring for eight days. The authors highlighted a significant reduction in the time required for sample analysis and a substantial 70% decrease in project costs compared to conventional tracer methods. Currently, the injection of a single CQD as a tracer has been expanded in Colombia in six different fields and 13 injection patterns [18], resulting in a cost reduction of about 90% regarding the projects using conventional tracers. 

These findings underscore the potential of carbon quantum dots as nanotracers, which can replace traditional ones by being applicable and stable under various petrophysical conditions. The increasing number of studies enhances knowledge about this technology, enabling its global and secure adoption in the oil industry. 

Although the findings from these applications are important, the primary limitation for achieving a comprehensive connectivity description is that more than one tracer is commonly required. Co-injecting tracers is frequently necessary to effectively visualize and quantify channeling effects or the impact of multiple injection wells. Consequently, applications thus far have been limited in their ability to fully leverage the technology in complex arrangements where only the injection of a single quantum dot is undertaken [15].

To the best of our knowledge, only a single carbon quantum dot has been used in simple patterns in the studies conducted to date. For complex well patterns, the most common scenario involves using multiple tracers with simultaneous quantification in the same effluents. Therefore, applications in which more than one carbon quantum dot is employed as a tracer remain challenging. 

Hence, the novelty of this study lies in the acquisition of diverse emission-length smart nanotracers and their application and simultaneous detection in the field. The investigation involves studying their interactions with rock and fluids, validating their safety for the reservoir under real conditions, and the subsequent field injection to demonstrate well connectivity. An additional contribution of this study is the obtention of carbon quantum dots (CQDs) from agro-industrial waste, suggesting an eco-friendly route for CQD production. This proposal significantly broadens the technological scope of nanotracers, particularly in the intricate applications of CQDs as interwell nanotracers.

## 2. Materials and Methods

### 2.1. Materials

Four different types of carbon quantum dots were employed. Three CQDs were purchased from Petroraza S.A. (Sabaneta, Colombia) and are labeled according to the “color” of the emission wavelength as CQDblue, CQDgreen, and CQDred. The fourth one was synthesized from orange peel (Nariño-Colombia) and labeled as CQDop. The cultivation area is located at an altitude of 2108 m above sea level, with an average temperature of 20 °C and a relative humidity of 85%. All other reagents used were of analytical grade. Ethylenediamine was obtained from Sigma Aldrich (Saint Louis, MO, USA), and ethyl acetate and urea were from Merck Millipore (Burlington, FL, USA). Production water and crude oil were obtained from Colombia’s central region oilfield. 

### 2.2. Nanotracer Synthesis

The orange peel nanotracer was synthesized using the hydrothermal carbonization methodology proposed by Sabet et al. [19] with some modifications. The collected oranges were washed twice with ultra-pure water and manually peeled; the organic material (orange peel) was carbonized at 180 °C for 20 min. Two grams of the carbonized peels were homogenized with 0.03 g/mL of urea and 0.06 g/mL of citric acid in 30 mL of distillate water. The solution was subjected to ultrasound for 20 min to homogenize the mixture. Finally, synthesis was conducted at 188 °C for 6 h in a hydrothermal autoclave reactor with a Teflon chamber. After the reaction, a homogeneous solution was obtained, filtered, and centrifuged at 6000 rpm for 20 min to remove insoluble residues. Finally, the supernatant was collected and stored at 4.0 °C for future analyses.

### 2.3. Nanotracers’ Characterization

#### 2.3.1. Physicochemical Characterization

Many techniques were employed for the physicochemical characterization of CQDs. Initially, Fourier transform infrared spectroscopy (FTIR) was conducted using an IRAffinity-1 FTIR instrument (Shimadzu, Kyoto, Japan) in transmittance mode with a resolution of 2 cm^−1^ over a range of 4000–400 cm^−1^ [20] to know the chemistry of the nanostructure. Dynamic light scattering (DLS) was used to measure the CQDs’ hydrodynamic diameter. To evaluate the stability of the hydrodynamic diameter measurement, measurements were made in distilled water, water with 0.9% NaCl, and the supernatant of the static retention test. Electrophoretic light scattering was employed for the determination of Zeta potential. Both tests were conducted using a NanoPlus Zeta/nanoparticle analyzer (Micromeritics, Norcross, GA, USA) at room temperature [21]. 

Morphological analysis was performed using transmission electron microscopy (TEM), and images were captured with a Tecnai G2 F20 microscope (FEI, Hillsboro, OR, USA) after preparing the sample using ultrasound to ensure good dispersion and obtain high-quality images. Finally, Atomic Force Microscopy (AFM) was employed to determine the surface roughness of the nanotracers using an AFM 5500 instrument (Agilent Technologies, Chandler, AZ, USA). The images were analyzed with the Gwydion 2.62 software, and the UV-visible absorption spectra of the CQDs were recorded using a Shimadzu UV 1800 UV spectrometer (Kioto, Tokyo, Japan) with 1 nm step readings and with standard Q Quartz glass 10 mm cuvettes, which have an optical path of 10 mm. Dispersions containing 100 mg/L of each of the nanotracers were used for the measurement.

#### 2.3.2. Fluorescence Measurements

A PerkinElmer LS-55 (Waltham, MA, USA) spectrofluorometer was used to determine the characteristic spectrum of the nanotracers [15]. Dispersions containing each nanotracer at a concentration of 100 mg/L were prepared using distilled water. These dispersions were introduced into the spectrofluorometer to establish the maximum excitation and emission wavelengths. These specific wavelengths are the fluorometric conditions essential for the distinct identification of each nanotracer. After confirming the specific excitation–emission conditions, calibration curves were meticulously crafted to illustrate the relation between concentration and fluorescence intensity emission. Solutions for the curve ranging from 1 to 500 mg/L were prepared by dissolving the nanotracer in distilled water. Subsequently, the fluorescence intensity of each solution was measured under the pre-determined specific excitation–emission conditions.

### 2.4. Fluid–Rock Interaction

To evaluate the interaction between rock (simulating reservoir mineralogy) and the CQD dispersions, a detergency test (Supplementary Materials), changes in the wettability, and static and dynamic retention tests were carried out as described below.

#### 2.4.1. Wettability Contact Angle

The wettability of porous media was determined by estimating the contact angle between a synthetic core and a drop of water before and after immersion in crude for 24 h dry and then in water with and without 100 mg/L of each CQD as described by Villegas et al. [22]. The test was conducted by depositing a water droplet on the surface of the restored and treated cores. The contact angle between the core and water is measured using an optical tensiometer Theta (Biolin Scientific, Västra Frölunda, Sweden) equipped with a high-definition camera.

#### 2.4.2. Static Retention Tests of Nanotracers

To evaluate the retention caused by the interaction between the rock and CQDs, a static test was carried out as described by Diez et al. [23] with some modifications. A batch-type adsorption setup with representative sand was used. Sand with 80% Quartz and 20% Kaolinite was prepared and washed with an ethanol/water solution to remove impurities and then dried in an oven. An amount of 20 g of washed sand was covered with 50 mL of CQD dispersion at 100 mg/L. The amount of adsorbed material was estimated by the difference in the concentration calculated at the beginning and after 24 h using the previously constructed calibration curves.

#### 2.4.3. Dynamic Retention Test

The dynamic test methodology was carried out following the approach proposed by Franco et al. [15]. To achieve the desired permeability, an artificial core was made from previously washed, dried, and sieved sand. The test conditions are shown in Table 1.

**Figure 1:**
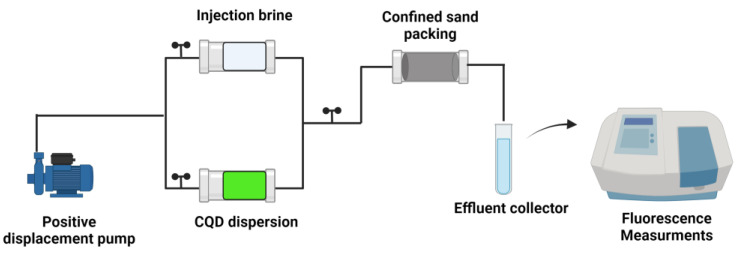
Schematic representation of the setup for the displacement tests.

The tracer injection was carried out following the setup represented in Figure 1. Effluents were collected to measure their fluorescence using the PerkinElmer Fluorometer. The data obtained allowed the construction of the nanotracer breakthrough curve.

### 2.5. Technology Assurance and Field Implementation 

#### 2.5.1. Baseline 

Before the injection stage, the natural fluorescence of produced water must be measured and periodically monitored. The natural fluorescence of the injection waters must be known. It will be used as a control parameter to establish the CQDs’ presence after injection. Fifteen days before the CQDs’ injection, water samples were collected from the production wells, and the fluorescence was analyzed in the laboratory.

#### 2.5.2. Conventional Tracer Fluorescence

During the performance of this study, a fluor benzoic acid tracer is injected into the field, so it is necessary to consider the fluorescence signal caused by this molecule. Conventional tracer solutions at 1–1000 mg/L concentrations were prepared and evaluated in the spectrofluorometer.

#### 2.5.3. Simultaneous Quantification

This study held a significant challenge: concurrently injecting multiple nanotracers utilizing carbon quantum dots. Consequently, before implementing these in the field, it was imperative to assess the feasibility of independently quantifying each carbon quantum dot (CQD), even in the presence of other nanotracers within the same dispersion. Four dispersions were prepared, each with different concentrations of the four nanotracers, as follows. Dispersions were prepared with the four nanotracers, each of them at different concentrations of 10, 50, 250, and 500 mg/L. The concentration of each CQD was measured in every dispersion. A comparison was then made between the prepared concentration and the concentration calculated using fluorometric calibration curves. This analysis aimed to ascertain the feasibility of quantifying each tracer in a mixture under the given conditions.

### 2.6. Field Implementation

The injection of CQDgreen occurred in January, and CQDblue was injected in March. A total of 50 gallons of aqueous dispersion, containing 71 kg of each CQD, was injected into wells I1 and I2, respectively. A 700 psi pump facilitated the injection process, with operations paused for tracer disposal before recommencing for movement. Over one year, the periodic monitoring of the effluents in the production wells took place. The collected samples underwent processing to extract water and were then analyzed to detect both CQDs. The baseline established before injection served as a comparison parameter, determining the presence of CQDs in the outlet fluids.

## 3. Results and Discussion

### 3.1. Fourier Transform Infrared Spectroscopy (FTIR)

**Figure 2:**
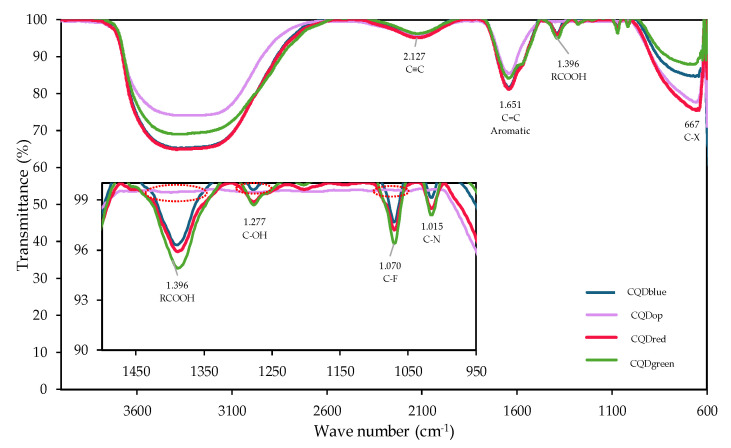
FTIR spectrum of commercial carbon quantum dots CQDblue, CQDgreen, and CQDred, and CQDop was synthesized from orange peel.

This technique determines CQDs’ functional groups. Figure 2 shows the FTIR analysis results for quantum tracer samples.

Several chemical groups in CQD samples were identified throughout the spectrum. According to Vieira et al. [24], the structure of a CQD is based on a core–shell shape that can be graphitic crystalline (sp^2^) or amorphous (mixed sp^2^/sp^3^), depending on the abundance of the occurrence of sp^2^ carbon in the core. Many functional groups related to the components used during the synthesis steps can be attached to the core. Given the nature of these nanoparticles, the presence of various carbon atoms bonded by triple bonds (2127 cm^−1^) [25] and forming cyclic structures with carbon–carbon double bonds (1651 cm^−1^) [26] was expected. The prevalence of unsaturations with sp configuration of triple bonds remains consistent across all the carbon quantum dots investigated. However, more pronounced picks in the CQDred FTIR spectrum exhibit more sp^2^ unsaturations. This unique optical behavior, as elucidated later, is intricately linked to the specific hybridization of electrons within the carbonaceous structure.

In addition to these common bands, it was possible to identify chemical groups present exclusively in the commercial tracers. Carboxylic groups (1396 cm^−1^) [27] and hydroxyl groups (1277 cm^−1^) [28] were detected. These two are strongly related to the materials’ negative charge surface (to be demonstrated later). Towards the end of the spectrum, the presence of halogenated groups was also observed (667 cm^−1^) [29].

The fundamental differences in the chemistry of the commercial materials compared to those synthesized according to the developed protocol can be directly associated with the synthesis method and the raw materials used for their production. While the detailed protocols of commercial products are unknown, the presence of atoms intercalating with the fundamental molecule, such as fluorine (1070 cm^−1^) and nitrogen (1015 cm^−1^) [28], is evident. These atoms modify the structure and behavior of the tracers, which is related to their fluorescence patterns discussed later. 

### 3.2. Zeta Potential and Dynamic Light Scattering (DLS)

The values obtained for the hydrodynamic diameter were 95.5 nm, 12 nm, 36 nm, and 37.4 nm for CQDgreen, CQDred, CQDblue, and CQDop, respectively. These measures were significantly greater than those evaluated by TEM microscopy. TEM is an observation of the individual nanoparticle, allowing for the edge of the CQD to be much more delimited. Authors such as Lim et al. [30] connect this phenomenon to the measurement of small aggregates of CQDs in aqueous dispersion using DLS. They suggest that factors such as sample concentration effects or limitations of the equipment, given the small size of the CQDs, could lead to an overestimation of the values of the hydrodynamic diameter of the CQDs. Measurements were also conducted in a 0.9% NaCl solution, where differences < 3% were obtained regarding distilled water with values of 94.8 nm, 12.1 nm, 36.9 nm, and 36.1 nm, respectively. These results suggest that the colloidal stability of the evaluated CQD suspensions is high. 

The zeta potential allows for identifying the surface charge of the CQDs and the electrostatic contact that may occur between them [31]. The magnitude of the zeta potential indicates the colloidal stability of a system. As mentioned by Larson et al. [32], the charge (negative or positive) of the zeta potential is associated with the surface charge of the nanoparticle in the dispersion at the medium’s conditions. Whether the value is positive or negative, a high magnitude of the zeta potential value is generally associated with a strong electrostatic repulsion between the nanoparticles, preventing them from coming into close contact and aggregating. Therefore, higher zeta potential values indicate greater stability against aggregation phenomena. The values obtained for the zeta potential of the CQDs under study were −49.09 mV, −21.79 mV, −17.80 mV, and −20.32 mV for CQDgreen, CQDred, CQDblue, and CQDop, respectively.

These results agree with Ateia et al. [33], who obtained CQDs from citric acid and orange juice using hydrothermal synthesis and chemical oxidation techniques. When evaluating the zeta potential of the synthesized CQDs, the authors observed negative values ranging between −21 and −30 mV. These values are attributed to the presence of oxygen-containing functional groups on the surface of the CQDs. Moreover, Clogston et al. [31] define CQDs as strongly anionic when their zeta potentials fall below −30 mV. The values obtained for the CQDs in this study support their anionic nature. This observation is related to the FTIR analysis, which reveals that CQDgreen exhibits a higher abundance of negatively charged chemical groups on its surface than the other samples.

### 3.3. Morphology (TEM-AFM)

**Figure 3:**
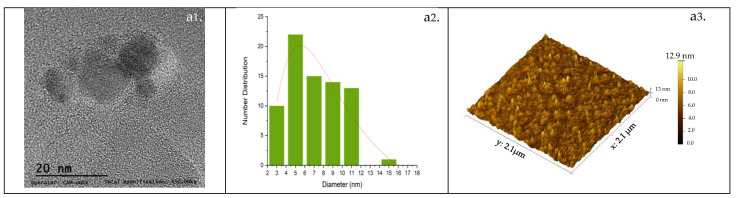

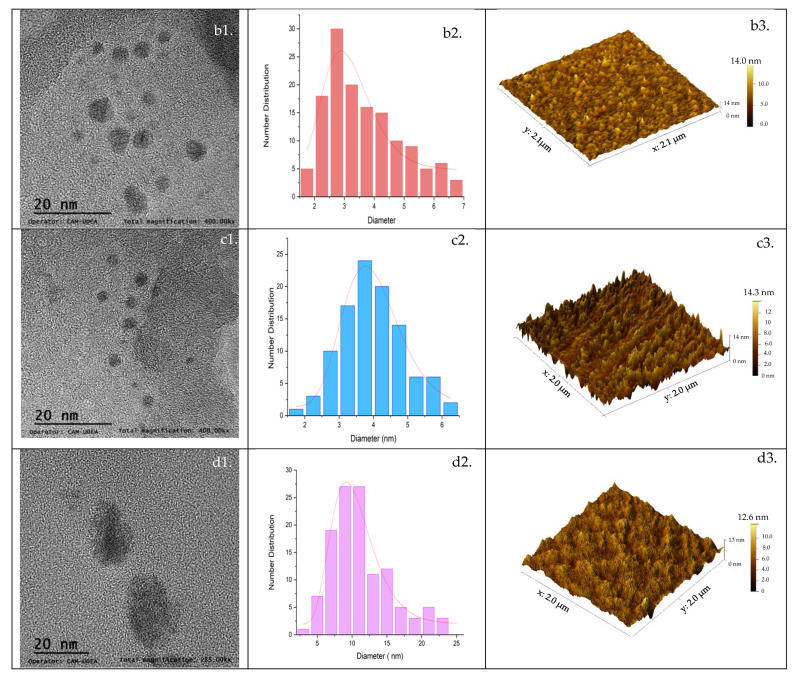
TEM images of (**a1**) CQDgreen, (**b1**) CQDred, (**c1**) CQDblue, and (**d1**) CQDop; average diameter histogram of (**a2**) CQDgreen, (**b2**) CQDred, (**c2**) CQDblue, and (**d2**) CQDop; and AFM images of (**a3**) CQDgreen, (**b3**) CQDred, (**c3**) CQDblue, and (**d3**) CQDop.

Transmission electron micrographs and atomic force microscopy were used to elucidate the morphology of the CQDs and the results are shown in Figure 3.

TEM micrographs allow us to determine the crystal morphology detected as parallel lines, forming round structures that confirm the presence of nanocrystals. Detailed analysis of the TEM images demonstrates a quite monodispersed and spherical morphology; these structures have also been seen in the CQDs analyzed by Su et al. [34], which revealed that the CQDs are zero-dimensional nanomaterials with diameters between 1 and 20 nm. The statistical analysis of the micrographs allows for the calculation of the average diameters of CQDgreen, CQDred, CQDblue, and CQDop as 8.5 nm, 5.7 nm, 3.9 nm, and 10.1 nm, respectively. The presence of constant irregularities on the surface of the CQDs can be seen in the AFM images. The average surface roughness was 3.1 nm, 2.1 nm, 2.9 nm, and 1.4 nm for CQDgreen, CQDred, CQDblue, and CQDop, respectively. The CQDs’ morphology was similar to the findings of Lim et al. [35] for carbon quantum dots synthesized using citric acid. These studies revealed a thickness in the range of 1–20 nm. Authors such as Islam et al. [36] relate these defects to imperfections or irregularities in the crystalline structure of the material, which are generated naturally during its formation and could affect its ability to emit photoluminescence.

### 3.4. Spectrophotometric Properties

**Figure 4:**
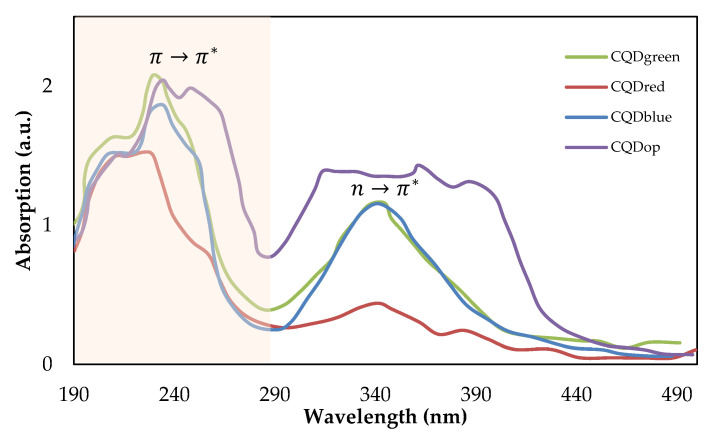
UV-Vis spectra of commercial carbon quantum dots CQDblue, CQDgreen, and CQDred and CQDop synthesized from orange peel.

The interactions between light and certain chemical compounds allow us to determine absorption patterns in the ultraviolet or visible regions [37]. These interactions have been the basis for the study of UV-visible spectroscopy. UV-visible absorption spectra were measured between 180 and 500 nm. Figure 4 shows the UV absorption spectrum of all the carbon quantum dots under study.

The bands observed between 200 and 300 nm correspond to electronic transitions of aromatic structures with C=C unsaturation of the π→π* type and bonds associated with functional groups containing oxygen present on the surface of the CQDs, and these are consistently detected in all synthesized CQDs. The absorption band observed at 340 nm is related to the n→π* electronic transition of the C=O bond present in the carboxylic acid [38,39]. The absorption bands agree with the functional groups determined in the FTIR analysis at wavenumber of 1651 cm^−^^1^ for the conjugated structures and 1396 cm^−^^1^ (related to the sp3 carbon) of the carboxyl group. Shapiro et al. [40] suggest that the location of absorption bands generally varies depending on the precursor and the synthesis technique.

**Figure 5:**
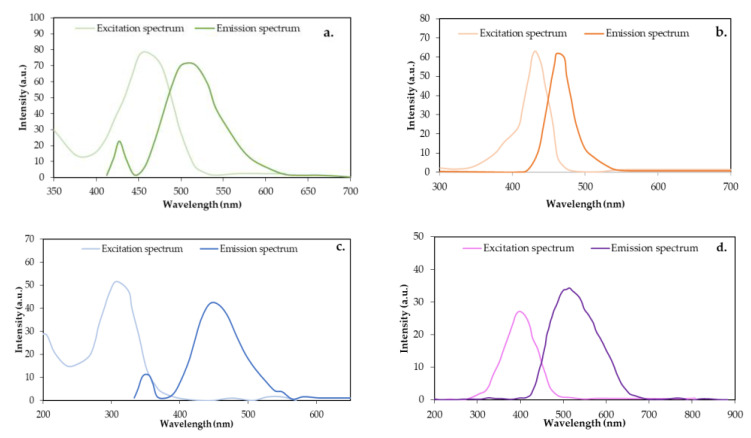
Fluorescence excitation and emission spectrum: (**a**) CQDgreen, (**b**) CQDred, (**c**) CQDblue, and (**d**) CQDop.

According to Lakowicz et al. [41], fluorescence is an optical phenomenon in which certain substances, called fluorophores, absorb light energy of a specific wavelength and subsequently emit it as longer-wavelength light. This process occurs when the electrons of the fluorophore atoms or molecules are excited to higher energy levels by absorbing photons. The fluorescence spectrum for the CQDs of the present study is shown in Figure 5.

The fluorescence spectra allow for the identification of the maximum excitation and emission lengths of the fluorescence pattern. The lengths where the maximum excitation–emission signals were found were 446–515 nm for CQDgreen, 550–577 for CQDred, 396–450 nm for CQDblue, and 407–420 nm for CQDop. A classification based on emission length [42] reveals that the analyzed CQDs correspond to cyan, yellow, blue, and violet light, respectively. Certainly, a single factor does not completely govern the mechanism by which carbon quantum dots can emit energy. Studies related to the synthesis of CQDs with unique optical properties describe factors such as the size [43], synthesis route [44], and precursors of the reaction [45]; passivation and surface coating [46]; as well as the presence of heteroatoms [47]. Variations in experimental factors and these parameters have allowed authors to obtain CQDs with emission lengths in a wide range (300–600 nm). Several anticipated theoretical explanations exist to elucidate the emissive characteristics of CQDs, including the quantum size effect, surface-state electron-hole radiation reorganization, and molecular-state luminescence emission mechanism [33]. Where the emission length shift is more toward the red, this shift is attributed to structures with a higher conjugation. Particularly for CQDred, which has a longer emission length (574 nm), a greater abundance of conjugated unsaturations is observed in aromatic structures. The maximum emission lengths of the other CQDs correspond to 420 nm for CQDop, 450 nm for CQDblue, and 515 nm for CQDgreen, a strong shift in this case, as sp^2^ hybridization allows for greater delocalization of the π electrons, which can favor the absorption and emission of light at shorter wavelengths, such as those corresponding to the color red. 

The emission lengths found and the average sizes measured by TEM do not have a clear correlation. Although the smallest CQD is blue (shortest emission length), for the other CQDs, the size and emission length vary independently; therefore, the differences in the fluorescence pattern of the CQDs studied could be more related to the chemical groups present on the surface of the nanocrystal than to the size effect. 

**Figure 6:**
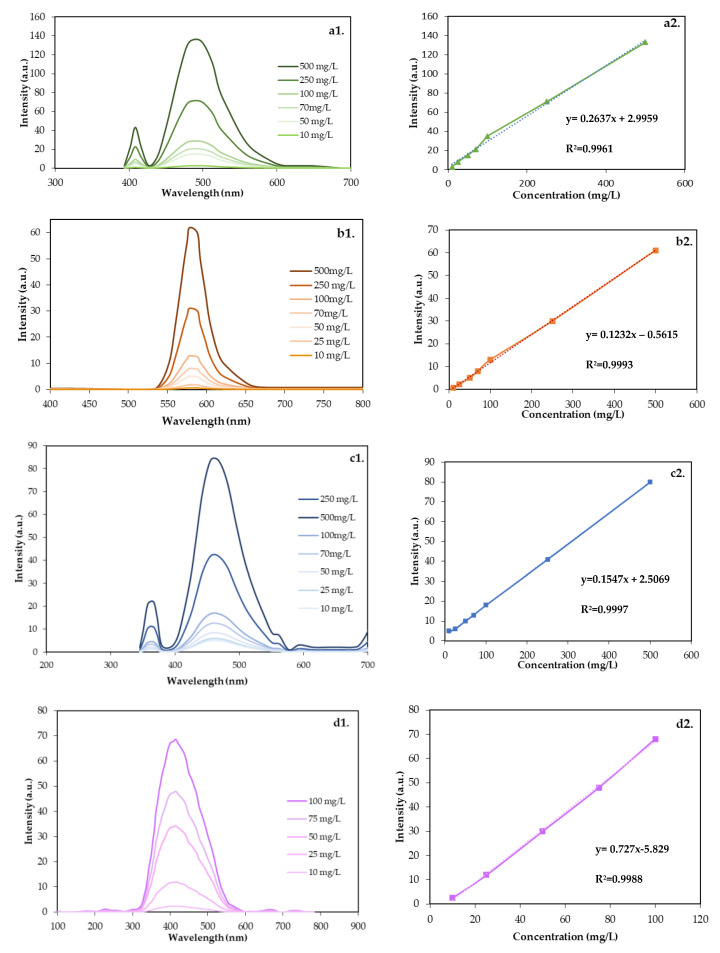
(**a1**) Emission spectra at different concentrations of CQDgreen; (**a2**) correlation of concentration and maximum emission intensity of CQDgreen. (**b1**) Emission spectra at different concentrations of CQDblue; (**b2**) correlation of concentration and maximum emission intensity of CQDblue. (**c1**) Emission spectra at different concentrations of CQDred; (**c2**) correlation of concentration and maximum emission intensity of CQDred. (**d1**) Emission spectra at different concentrations of CQDop; (**d2**) correlation of concentration and maximum emission intensity of CQDop.

Once the optimal excitation and emission conditions have been identified, it is crucial to verify the existence of a linear correlation between the fluorescence intensity measurements under these conditions and the concentration of a sample containing carbon quantum dots (CQDs). Figure 6 shows the evaluated intensity–concentration relationship between 10 and 500 mg/L for each nanomaterial evaluated.

The relationship between concentration and intensity shows linearity for each of the CQDs, with correlation coefficients (R^2^) up to 0.99 in all cases. Selecting a specific excitation–emission pair for each CQD increases the specificity of the measurement. The radiated energy is expected to be selectively absorbed by the molecular structures capable of doing so at that wavelength and that are present in the nanoparticle, excluding others in the surrounding environment [48]. This improves sensitivity, specificity, and substance identification, resulting in more accurate and reliable measurements. 

Henceforth, the developed curves will be used to determine the concentration of the nanotracer in unknown samples. This approach allows for quantification by linear interpolation using the specific tracer curve to be quantified.

### 3.5. Contact Angle Measurements

Changes in the wettability of the porous media were also measured [49]. This property is crucial because it can affect the efficiency of the enhanced oil recovery processes. If the rock is water-repellent, displacing oil through water injection can be challenging. In this investigation, CQDs were not designed to change wettability behavior. However, it is important to measure it as a control and technological assurance parameter and as an indirect measurement of retention in the porous medium.

Wettability was calculated using the contact angle between a water droplet and a solid surface at the point of contact. The contact angle values for the water in the absences of nanomaterials and the cores that have been in contact with CQD dispersions at 100 mg/L were as follows: 79.5° for the control sample, 78° for CQDgreen, 79.0° for CQDred, 76.5° for CQDblue, and 78.9° for CQDop. A marginal reduction in the contact angle was observed across all instances. These small changes suggest that there are no negative effects in wettability when injecting the CQDs and that the nanomaterials can perform in an adequate way as interwell tracers. 

#### 3.5.1. Static Retention Tests

**Figure 7:**
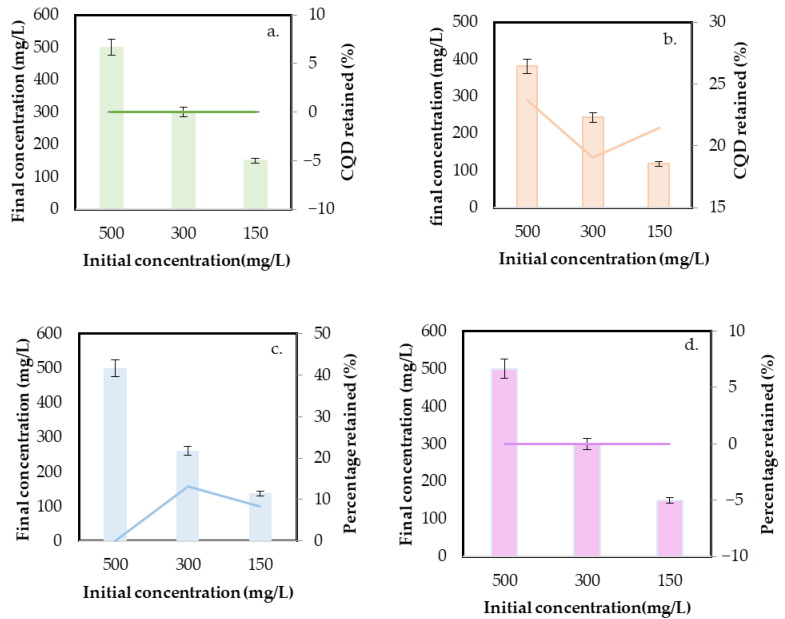
Static retention test in 80% Quartz–20% Kaolinite sand and dispersions with an initial concentration of 100mg/L for (**a**) CQDgreen, (**b**) CQDred, (**c**) CQDblue, and (**d**) CQDop. The CQD retained (%) was estimated by the difference in the concentration calculated at the beginning and after 24 h. The mean deviation values correspond to <10% of the measurement.

The static retention test indicates the degree of interaction of the CQDs with the porous medium and provides insight into the amount of tracer that could remain retained [50]. The results are presented as the retained concentration for each CQD and the percentage regarding the initial concentration in dispersion. The obtained results are shown in Figure 7.

The results indicate that CQDgreen, CQDblue, and CQDop exhibit no retention within the porous medium. Conversely, for CQDred, a retention phenomenon within the porous medium is evident, independent of the initial nanotracer concentration in the solution. On average, 21.4% of the introduced CQDred was retained. Sun et al. [51] studied the transport and retention of CQD synthesized using a hydrothermal route, affirming that the retention in saturated and unsaturated porous media is related to ionic strength and sand grain size. According to the authors, retention increases with the increased ionic strength because of the reduction in electrostatic repulsion. Due to the studies being conducted with a representative rock, this outcome signifies that in media primarily composed of Quartz and Kaolinite, an undesirable retention phenomenon could occur with the CQDred, conflicting with the intended application. 

The size of all four CQDs was analyzed through DLS after retention, with changes in the average hydrodynamic diameter < 3% regarding the size before the tests (see Section 3.2). This result corroborates the high stability of the selected nanomaterials under the conditions evaluated. 

#### 3.5.2. Dynamic Retention Tests

**Figure 8:**
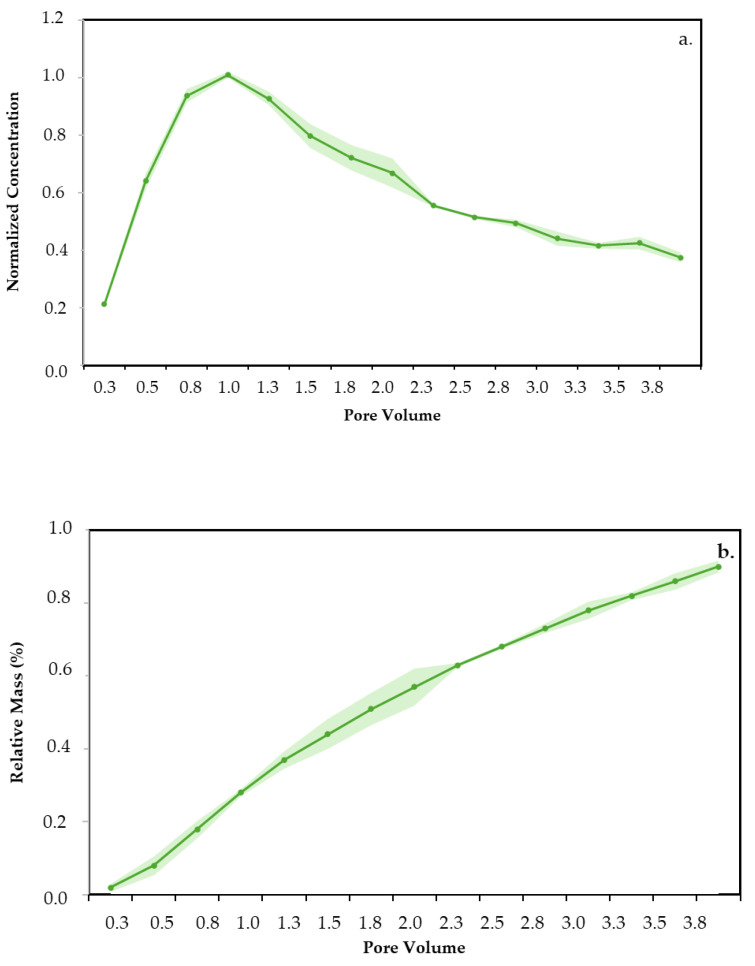
Dynamic retention tests of CQDgreen: (**a**) injection breakthrough and (**b**) mass balance.

Only one of the nanomaterials was selected for the test. Due to similarities in static retention with CQDblue and CQDop, CQDgreen was chosen based on possibilities for scaling up for a field trial. The injection breakthrough curves of CQDgreen and its corresponding mass balance measured through detection in the effluents are shown in Figure 8. 

The dynamic test results demonstrate that CQDgreen has no retention within the porous medium, aligning consistently with the static test outcomes. The maximum tracer concentration value is found after a 1.0 porous volume of injection, showing a piston-type flow behavior capable of moving completely and without restrictions through the porous medium. In addition, more than 95% of the total injected mass of the nanotracer is recovered. This rapid decline in concentration strongly suggests that the introduced tracer swiftly departs the formation upon contact, exiting the porous medium entirely.

### 3.6. Technology Assurance and Field Application 

#### 3.6.1. Baseline Construction

**Figure 9:**
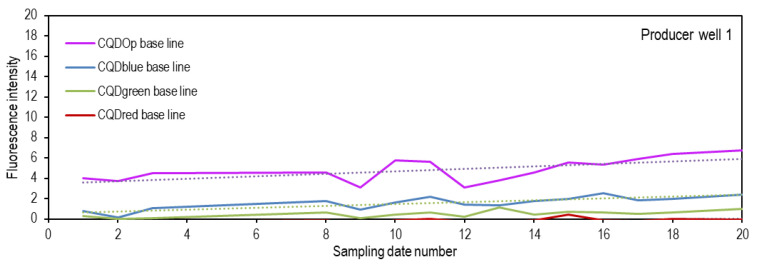
Baseline fluorescence. Natural fluorescence of the production water for the producing well 1 measured at the conditions of each of the CQDs. Purple line: fluorescence reading at excitation and emission lengths of CQDop. Blue line: fluorescence reading at excitation and emission length of CQDblue. Green line: fluorescence reading at excitation and emission length of CQDgreen. Red line: fluorescence reading at excitation and emission length of CQDred. Dashed lines represent the linear trend of florescence measured over time.

The baseline was constructed for each of the wells considered in the interwell connectivity study, and spectrofluorometric measurements were taken according to the previously established conditions for each CQD. Sampling was conducted on twenty different dates before the injection of the CQDs to estimate the natural fluorescence of the production waters. This approach will allow for the confirmation of the tracer’s presence in the evaluated well when the fluorescence signal exceeds the average of the natural signal plus three times the standard deviation of the measurements made before the CQDs’ injection. Figure 9 shows the baseline for one of the wells under investigation. However, it is worth mentioning that the same procedure was carried out for all wells within the influence zone.

According to the findings depicted in Figure 9, the intrinsic fluorescence of the water sample consistently registers at levels below ten across all assessed dates under the parameters of all CQDs. This consistent trend suggests that the introduced chemical agents and inherent constituents within the production water do not manifest a substantial fluorescence signal when analyzed under the specifications of the CQDs’ conditions. Comparable patterns were observed in the remaining pertinent producing wells (data not presented).

#### 3.6.2. Simultaneous Quantification 

This experiment simulates a scenario where four CQDs are present in the same sample. The results of the individual quantification of the four CQDs are shown in Table 2.

As observed in the results, despite having four CQDs present simultaneously in the dispersion, the calibration curves allowed for a quantification of each CQD very close to the theorical values, with deviations of 11.55%, 14.32%, 7.90%, and 15.48% for CQDgreen, CQDred, CQDblue, and CQDop, respectively. Despite a slight deviation in the measurement when the CQDs were present simultaneously compared to the individual dispersions, it is possible to quantify them with a good approximation. Each CQD has an independent linear relationship, responding to its concentration and the fluorescence intensity read at specific conditions.

Also, the conventional tracer does not exhibit a significant fluorescence intensity within the evaluated concentration range. Consequently, when assessing the intensity values on the calibration curve for each CQD, the concentrations were equal to zero. These results suggest that the presence of the conventional tracer will not generate a significant fluorescence signal that could interfere with the quantification of any of the CQDs if any of them coexist with the traditional tracer.

### 3.7. Field Implementation

Post-assurance at a laboratory scale, CQDgreen and CQDblue were selected for the field implementation. According to the results, the connectivity between the wells would depend exclusively on the CQD detected in the effluents. The time required for its appearance is correlated with the injector–producer well distance. The tracking and analysis of the field samples took over one year. 

**Figure 10:**
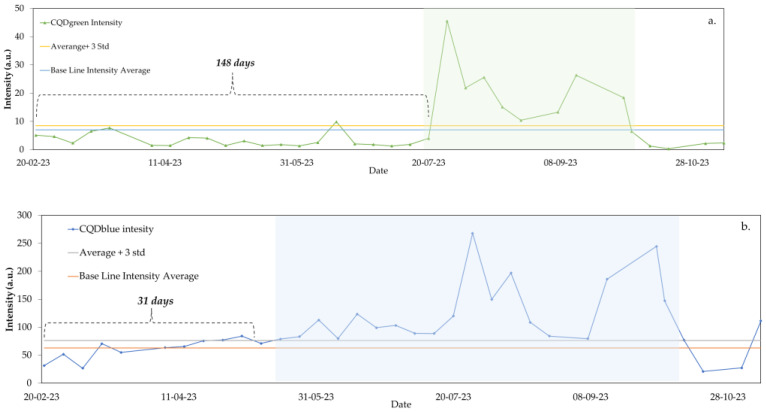
First nine months of tracer tracking: (**a**) CQDgreen and (**b**) CQDblue monitoring in Producer Well 1.

The main difficulty in monitoring the nanotracer is the absence of water in some of the effluents taken. Given that the fluorescence analysis had to be conducted in an aqueous portion, some of the samples taken for the monitoring and detection of the nanotracers were discarded since they consisted only of crude oil. However, the samples where water was obtained were analyzed. A historical record of the injected CQD concentration was created for each well under study. The historical record of one production well is described in Figure 10.

**Figure 11:**
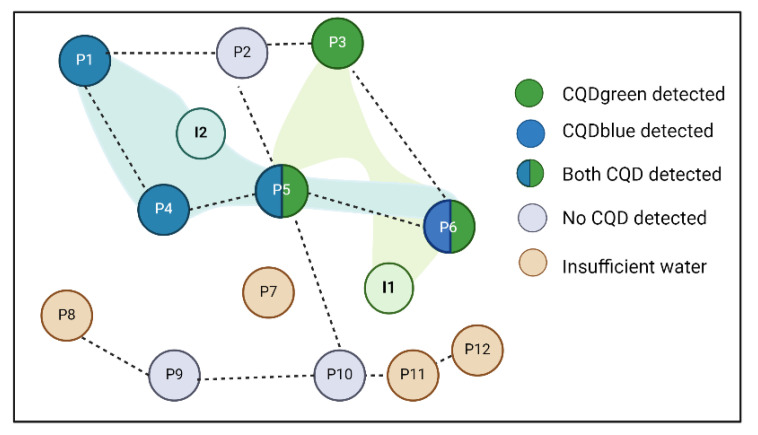
Interwell connectivity determination with CQDs.

As observed in Figure 10, both CQDs were effectively tracked and quantified in real-time within the production waters. The detection of CQDgreen was noted in the fourth month after its injection and was persistently detected for a consecutive three-month period. Conversely, the blue tracer manifested one month after its injection and remained detectable for six months. The proximity of the production well to the injection wells is consistent with the timing of the appearance of each tracer in the effluents. A similar analysis was conducted for each production well; a primary description is shown in Figure 11.

To date, the fluid flow pattern in the northern zone is completely described, where some wells are only influenced by injection well two and others by both. The scope of influence of injection well one was unknown, and the information derived from this study is important, especially for the planning of future enhanced recovery projects in the studied pattern. Prior to the monitoring period, the injection of two conventional tracers (fluorobenzoics) and two nanotracers was conducted in four inverted 5-point patterns. As of the current stage, the conventional tracers have not yet been detected during the monitoring, whereas the monitoring of the CQDs has been carried out. Given their rapid response times and analytical precision, the study remains ongoing to achieve a more thorough and detailed characterization of the area. Nonetheless, these preliminary findings are closely aligned with geological studies and the spatial arrangement of wells within the pattern.

The carbon quantum dots employed in this study are devoid of heavy metals known for their environmental toxicity. However, addressing environmental concerns related to quantum dots remains intricate due to the diverse nature of this nanomaterial category. Quantum dots encompass a broad and heterogeneous range of nanomaterials, distinguished by variations in size, charge, concentration, coating, surface modifications, oxidative stability, and optical properties. Consequently, determining their environmental implications, including absorption, distribution, metabolism, excretion, and toxicity, hinges upon numerous factors stemming from intrinsic physicochemical properties and environmental conditions. Thus, comprehensive and targeted investigations are imperative to ascertain the environmental safety of these nanomaterials.

## 4. Conclusions

This study proposes an efficient and successful application of carbon quantum dots as fluid flow descriptors in a complex reservoir. The transition from a laboratory scale to field implementation allows for the positioning of the technology as feasible and has promising results. To achieve the above, an eco-friendly protocol for synthesizing nanotracers using agro-waste such as orange peels as the main precursor was developed. The chemical, physical, morphological, and optical properties were comparable with commercial CQDs that were included in the study. It was found that for all the CQDs evaluated, the diameter was between 3.4 and 10.1 nm, with a zero-dimensional spherical shape.

The fluid–rock interactions revealed that the CQDs studied do not alter or modify the porous medium. The natural properties of water remain unaltered by the presence of the nanotracer particles. Moreover, protocols for the individual quantification of the nanotracers, even in scenarios with multiple tracers, are proposed. The optical behavior of these nanoparticles is inherent to their chemical nature and remains unmodified by the presence of other particles. Therefore, it is feasible to implement them in fields with complex arrangements of injection and production wells. 

Although the carbon quantum dots used in this work were not synthesized using heavy metals and they are presumed to be environmentally harmless, detailed studies of their ecotoxicity should be developed to evaluate the possible environmental implications.

The sample tacking during the study period allowed us to identify some of the main interwell connections, and the time and well in which the nanotracer was present was consistent with the geological studies. These preliminary results represent an important growth for nanotracer technology and propose a safe and eco-friendly alternative. 

## Figures and Tables

**Table 1:** Reservoir operating conditions for the dynamic test.

Parameter	Value
Porosity	20%
Mineralogy	80% Quartz and 20% Kaolinite
Pressure	2000 psi
Temperature	114 °C
SW	22%
Injection water salinity	800 ppm

**Table 2:** Simultaneous quantification results of the tracers.

Tracer	Theoretical Concentration (mg/L)	Calculated Concentration (mg/L)	Interference Percentage (%)
CQDgreen	500	567.16	11.55
	250	285.47	
	50	54.33	
	10	9.01	
CQDred	500	550.10	14.32
	250	281.02	
	50	58.45	
	10	11.80	
CQDblue	500	538.42	7.90
	250	246.71	
	50	47.77	
	10	11.81	
CQDop	500	468.21	15.48
	250	267.78	
	50	57.10	
	10	13.43	

## Data Availability

Data are contained within the article and Supplementary Materials.

## Supplementary Materials

The following supporting information can be downloaded at: https://www.mdpi.com/article/10.3390/nano14090789/s1, Table S1. Crude oil-water Compatibility test using 20:80, 50:50 and 80:20 proportions of each of the phases, photographic record at the beginning and after 2 h at reservoir temperature. Figure S1. Results of the detergency test after 1 h at 80 °C using 80% Quartz, 20% Kaolinite as solid phase and solutions with 100 mg/L of a. CQDgreen, b. CQDred, c. CQDblue, and d. CQDop. Refs. [15,52,53,54] are cited in the supplementary materials.
