# Supplementary material for: Simultaneous Detection of Carbon Quantum Dots as Tracers for Interwell Connectivity Evaluation in a Pattern with Two Injection Wells
Source: Nanomaterials (Basel). 2024 May 1;14(9):789. doi: 10.3390/nano14090789 (PMC11085186; doi:10.3390/nano14090789)
Supplement: Supplementary file 1 [file nanomaterials-14-00789-s001.zip › nanomaterials-2954058-supplementary.pdf]

nanomaterials

Supporting information

Simultaneous Detection of Carbon Quantum Dots as Tracers for Interwell Connectivity Evaluation in a Pattern with Two Injection Wells

Stephania Rosales [1], Karol Zapata [1], Farid B. Cortes [1,*], Benjamín Rojano [2], Carlos Diaz [3], Carlos Cortes [3], David Jaramillo [4], Adriana Vasquez [4], Diego Ramirez [4] and Camilo A. Franco [1,*]

[1] Grupo de Investigación en Fenómenos de Superficie–Michael Polanyi, Facultad de Minas, Universidad Nacional de Colombia, Sede-Medellín, Medellín 050034, Colombia; srosalesd@unal.edu.co (S.R.); kzapata@unal.edu.co (K.Z.)
[2] Grupo de Investigación Química de los Productos Naturales y los Alimentos, Facultad de Ciencias, Universidad Nacional de Colombia, Sede-Medellín, Medellín 050035, Colombia; brojano@unal.edu.co
[3] GeoPark Colombia SAS, Bogotá 111211, Colombia; cdiaz@geo-park.com (C.D.); ccortes@geo-park.com (C.C.)
[4] Verano Energy Limited Sucursal, Bogotá 110211, Colombia; adriana.vasquez@parexresources.com (A.V.)
* Correspondence: fbcortes@unal.edu.co (F.B.C.); caafrancoar@unal.edu.co (C.A.F.)

S1. Content

S1.1. Compatibility Test

For compatibility test, the method proposed by Franco et al. [1] with some modifications was made: dispersions using 100 mg/L of CQD were prepared in injection brine, different volumetric relations were prepared using the CQD dispersions: crude oil, i.e., 20:80, 50:50 and 80:20 for a total volume of 10 mL, the mixes were agitated for 30 s and stored at reservoir temperature (237°F) for 2 hours, then the mixtures were analyzed to see if an emulsion or other phases had formed (incompatibility).

S1.2. Detergency Test

For detergency test, the protocol proposed by Cutler et al. [2] with some modifications was used; representative reservoir sand (80% Quartz, 20% Kaolinite) was soaked in 20 mL of treatment solution (Brine with 100mg/L CQD) for 1 hour at reservoir temperature. Subsequently, the sand was removed from the soak, and excess treatment was eliminated. Then, the soaked material was mixed with crude oil and water in a 50/50 ratio. Finally, the system was agitated, and the mixtures were left to soak at reservoir temperature for 1 hour. This test aims to inspect the sand's appearance in each container, establishing acceptable or unacceptable detergency based on the sand's coloration.

S2. Results

The compatibility test was conducted for each CQDs using crude oil and formation water to assess the nano tracer interaction scenarios fully when included in the injection waters. Table S1 shows the test results.

Table S1. Crude oil- water Compatibility test using 20:80, 50:50 and 80:20 proportions of each of the phases, photographic record at the beginning and after 2 hours at reservoir temperature.

Sample	Time = 0h	Time = 24h
CQDgreen	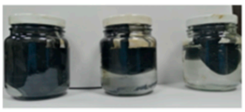	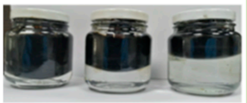
CQDred	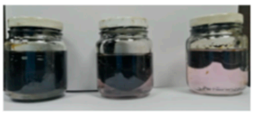	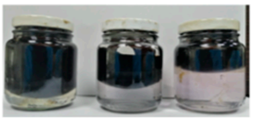
CQDblue	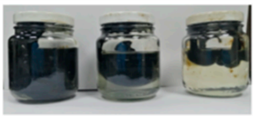	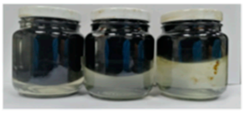
CQDOp	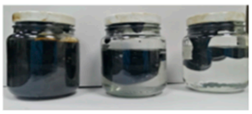	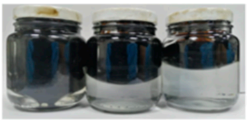

The photographic record of the compatibility tests carried out with crude oil from the formation and field water containing 100 mg/L of each nano tracer was carried out at the initial time of the test and after its agitation and exposure to reservoir temperature. For 2 hours, the samples were inspected to see if emulsions or aggregations formed in the phases brought into contact. In none of the cases did carbon quantum dots promote undesirable interactions between crude oil and water; the formation of undesirable phases, emulsions or aggregations was not observed. These results assure us that the technology to be implemented is safe in avoiding undesirable interactions or altering the stability of the natural fluids.

The interaction between the representative mineralogy of the formation and the fluid containing the CQDs was inspected through a detergency test carried out with injection water dispersions containing 100 mg/L of each CQDs. The photographic record of the porous medium and the fluids are presented in Figure S1.

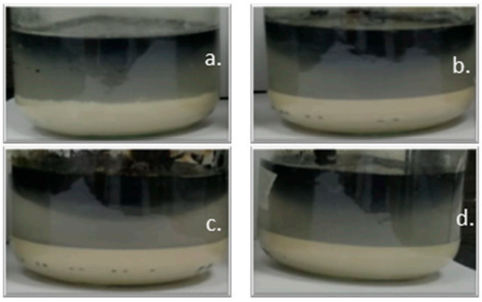

Figure S1. Results of the detergency test after 1 hour at 80°C using 80% Quartz, 20% Kaolinite as solid phase and solutions with 100 mg/L of a. CQDgreen, b. CQDred, c. CQDblue, and d. CQDop.

Observing the samples from the detergency test guarantees that the CQDs will not promote the retention of crude oil in the porous medium since each component is distinguished in its separated phases. Nor is there a detergent effect, foam formation or undesirable phenomena from the point of view of stability. The functional groups on the

surface of the CQDS may allow them to predominantly interact with the aqueous phase, forming hydrogen bonds that keep them dispersed in the water and prevent them from moving into the porous medium [3]. According to Serres et al. [4], from an operational approach, it is desirable that a tracer interact as little as possible with the porous medium and not promote undesirable interactions between water and crude oil. The results showed that none of the CQDs exhibited this behavior.

References

1. Franco, C.A.; Candela, C.H.; Gallego, J.; Marin, J.; Patiño, L.E.; Ospina, N.; Patiño, E.; Molano, M.; Villamil, F.; Bernal, K.M. Easy and rapid synthesis of carbon quantum dots from Mortino (Vaccinium Meridionale Swartz) extract for use as green tracers in the oil and gas industry: Lab-to-field trial development in Colombia. *Industrial & Engineering Chemistry Research* **2020**, *59*, 11359-11369.
2. Cutler, G. *Detergency: theory and technology*; CRC Press: 2020.
3. Shi, G.; Wang, H.; Zhang, Y.; Cheng, C.; Zhai, T.; Chen, B.; Liu, X.; Jono, R.; Mao, X.; Liu, Y. The effect of water on colloidal quantum dot solar cells. *Nature Communications* **2021**, *12*, 4381.
4. Serres-Piole, C.; Preud'Homme, H.; Moradi-Tehrani, N.; Allanic, C.; Jullia, H.; Lobinski, R. Water tracers in oilfield applications: Guidelines. *Journal of Petroleum Science and Engineering* **2012**, *98*, 22-39.
